# Illinois State Veterinary Medical Association

**Published:** 1901-12

**Authors:** J. J. Hughes, W. H. Welch

**Affiliations:** President; Secretary


### ILLINOIS STATE VETERINARY MEDICAL ASSOCIATION.

The Association held its nineteenth annual meeting at the Sherman House, Chicago, November 14 and 15, 1901.

The meeting was called to order by President T. J. Gunning at 10.30 A.M.

The following members were present during the meeting: Drs. T. J. Gunning, Neponset; F. H. Ames, Canton; V. E. Frizzelle, La Moille; N. I. Stringer, Watseka; H. A. Pressler, Fairbury; C. G. Glendenning, Clinton; W. B. Lewin, Russell; George B. Jones, Sidell; F. W. Kee, Sheldon; James Smellie, J. F. Ryan, A. C. Worms, C. F. Grinier, R. G. Walker, A. H. Baker, Jos. J. Hughes, Jas. Robertson, E. L. Quitman, all of Chicago; J. T. Nattress, Delevan; F. B. Rowan, Belvidere; W. J. Martin, Kankakee; R. F. Hoadley, Yorkville; Albert Babb, Springfield; John S. Spangler, Aurora; R. C. Mylne, Aurora; E. S. Fry, Naperville; Clarence Mills, Decatur; J. H. Crawford, Harvard; W. H. Welch, Lexington. Visitors: Dr. W. W. Welch, Elgin; Mr. H. A. Cole, of De Vaux Chemical Company; Mr. V. C. Barker, of Pasteur Chemical Company.

The minutes of the last meeting were read and approved.

The following names were presented for membership, and on motion were duly elected:

Dr. C. G. Glendenning, Ontario Veterinary College. Vouchers: W. H. Welch and T. J. Gunning.

Dr. Jos. Smellie, Chicago Veterinary College. Vouchers: E. L. Quitman and T. J. Gunning.

Dr. W. B. Lewin, Chicago Veterinary College. Vouchers: W. H. Welch and V. E. Frizzelle.

Dr. F. W. Kee, McGill Veterinary College. Vouchers: N. I. Stringer and H. A. Pressler.

Dr. J. H. Crawford, McKillip Veterinary College. Vouchers: R. G. Walker and Jas. Robinson.

Dr. Geo. B. Jones, Ontario Veterinary College. Vouchers: T. J. Gunning and E. L. Quitman.

Dr. F. B. Rowan, Chicago Veterinary College. Vouchers: N. P. Whitmore and E. L. Quitman.

On motion the Society adjourned until 1.30 P.M.

Immediately upon calling to order Dr. Gunning delivered the President's Annual Address.

Dr. E. S. Fry, of Naperville, now read his paper on "Azoturia." This subject brought out the usual lengthy discussion, and was participated in by Drs. Martin, Quitman, Pressler, Glendenning, Lewin, Jones, Mills, Stringer, Nattress, Ryan, Baker, Newby, and Hughes. A new feature in the discussion of this disease was the theory of germ origin.

Dr. T. B. Newby, of McKillip Veterinary College, now read his paper on "The Etiology of Omphalitis."

Dr. Newby maintained that quite a number of these cases, as he had proved by post-mortem, were of intra-uterine infection.

Discussed by Drs. Stringer, Hughes, Martin, Mills, Welch, and Baker.

Dr. Ryan now read his paper on "Trichina Spiralis and the Hog."

The discussion of this paper was postponed until Friday afternoon, and was participated in by Drs. Baker, Quitman, Grinier, and Mills.

On account of the clinics to be held at the Chicago Veterinary College from 9 A.M. until 12 M. the Society adjourned until Friday, at 1.30 P.M.

Friday morning the majority of members availed themselves of the opportunity of attending the clinics. These were varied, useful, and instructive.

Dr. Hughes, who was on the programme for a paper on "Quittor and its Treatment," took this opportunity to illustrate the surgical treatment as practised by himself.

Two or three quittors were operated on, and from one of them the lateral cartilage was removed. The Association now accepted the hospitality of Dr. Hughes for luncheon, after which they again met and witnessed a great many different operations performed by the doctor. Among these were mesoneurectomy, the high and low plantar neurectomies, operation for stringhalt, and the operation for cribbing, and caudal myotomy in all its forms.

It was nearly 4 P.M. when the Society reconvened at the Sherman House.

Dr. Hughes made a few remarks on "Quittor," and was excused.

Dr. Babb having been obliged to return home, his paper was held over until the next meeting.

The election of officers now took place, and resulted as follows:

President, Dr. J. J. Hughes, Chicago.

Vice-President, Dr. H. A. Pressler, Fairbury.

Secretary, W. H. Welch, Lexington.

Treasurer, R. G. Walker, Chicago.

Board of Censors, Dr. N. I. Stringer, Watseka; Dr. J. T. Nattress, Delevan; Dr. A. H. Baker, Chicago.

A vote of thanks was extended to the Chicago Veterinary College, also Dr. Hughes, for the magnificent clinics.

Dr. Hughes now took the chair and thanked the Association for the honor bestowed upon him.

Dr. Ryan moved a vote of thanks to the retiring officers.

President Hughes appointed the following committees:

Programme, E. L. Quitman, N. I. Stringer, and J. T. Nattress.

Arrangements, Jas. Robertson and W. J. Martin.

Legislation, Albert Babb, T. J. Gunning, and W. J. Martin.

Drs. Quitman and Baker spoke on the subject of trying to secure the next meeting of the American Veterinary Medical Association.

It was moved and carried that the Illinois State Veterinary Medical Association, through its officers, extend an invitation to the American Veterinary Medical Association to hold its next meeting in Chicago, and that the Secretary be instructed to solicit funds for entertainment.

The following amendments to the Constitution and By-laws were offered, and will be acted on at the next meeting:

Article II., Section 1—

*Resolved*, That Article II., Section 2, be amended to read "December" instead of "November." Signed: A. H. Baker and Robert G. Walker.

Also amending Article II., Section 1, to read as follows: "The meetings of the Association shall occur annually in Chicago during December, at such time as may be designated by the President and Secretary." Signed: C. G. Glendenning, W. H. Welch, and W. B. Lewin.

A vote of thanks was given the Sherman House for use of room.
Society adjourned to meet in Peoria in February, at call of President.

J. J. HUGHES, President. W. H. WELCH, Secretary.

---

